# Continuous Cropping of *Tussilago farfara* L. Has a Significant Impact on the Yield and Quality of Its Flower Buds, and Physicochemical Properties and the Microbial Communities of Rhizosphere Soil

**DOI:** 10.3390/life15030404

**Published:** 2025-03-04

**Authors:** Zhenbin Huang, Xia Wang, Liangshuai Fan, Xiaojun Jin, Xiang Zhang, Hongyan Wang

**Affiliations:** 1 College of Agronomy, Gansu Agricultural University, Lanzhou 730070, China; huangzb@st.gsau.edu.cn (Z.H.); wangx@st.gsau.edu.cn (X.W.); 1073323120614@st.gsau.edu.cn (L.F.); 1073323120618@st.gsau.edu.cn (X.Z.); wanghongy@st.gsau.edu.cn (H.W.); 2 State Key Laboratory of Arid Habitat Crops, Gansu Agricultural University, Lanzhou 730070, China

**Keywords:** *Tussilago farfara* L., continuous cropping years, soil, microbial community structure

## Abstract

Continuous cropping obstacles pose significant constraints and urgent challenges in the production of *Tussilago farfara* L. This experiment investigated the effects of consecutive cropping on *T. farfara* over periods of 1, 2, and 3 years. It assessed the yield and quality of *T. farfara* flower buds, in addition to the physicochemical properties of the rhizosphere soil. The microbial community in the rhizosphere was analyzed through 16S rDNA and ITS sequencing using Illumina Novaseq high-throughput sequencing technology, while also examining the correlations among these factors. The results reveal that as the duration of continuous cropping increases, the yield of *T. farfara* flower buds, along with the contents of extract, tussilagone, and total flavonoids, steadily decreased; soil pH, organic matter, available phosphorus, available potassium, alkaline nitrogen, and the activities of sucrose, catalase, and alkaline phosphatase markedly decreased. As the duration of consecutive cropping increases, the quantity and diversity of bacteria in the rhizosphere soil initially increase and then decrease, while the number of fungal species increases by 22.5%. Meanwhile, continuous cropping of *T. farfara* contributes to a gradual reduction in the relative abundance of beneficial genera such as Ralstonia, Nitrospira, and Trichoderma in the rhizosphere soil, while harmful genera such as Mortierella, Fusarium, and Tricharina accumulate significantly. Correlation analysis shows that changes in microbial communities notably influence the growth of *T. farfara* and soil quality. This study elucidates the impacts of continuous cropping on the yield and quality of *T. farfara* flower buds, soil physicochemical properties, and the microbial communities in the rhizosphere, providing a scientific basis for further research on continuous cropping barriers and the selection of beneficial microbial genera for the growth of *T. farfara*.

## 1. Introduction

*Tussilago farfara* L., a perennial herb belonging to the Asteraceae family, has dry flower buds that are utilized for medicinal purposes [1,2]. These buds primarily contain sesquiterpenes, flavonoids, sterols, and other bioactive compounds [3,4]. Pharmacological research indicates that *T. farfara* possesses several therapeutic functions, including cough suppression, lung protection, phlegm resolution, and anti-inflammatory effects [5]. However, due to overharvesting and the depletion of wild resources in recent years, this species is now sporadically distributed. Furthermore, artificial cultivation presents challenges, as the production process demands extensive manual labor—such as digging, sorting, bud picking, and drying—leading to high production costs, and it is both time-consuming and labor-intensive. Consequently, the area dedicated to artificial cultivation has decreased annually [6].

Continuous cropping obstacles refer to the degradation of soil quality, reduced crop yields, and increased occurrences of pests and diseases resulting from the repeated cultivation of the same crop in the same plot [7,8]. Research by Ku Y et al. has revealed that long-term continuous cropping adversely impacts the soil environment, crop growth, and fruit quality of cucurbits [9]. Fujisao K et al. discovered that corn yields decline annually as the duration of continuous cropping extends [10]. Yao Q et al. found that for alfalfa, within the first 10 years of continuous cropping, soil moisture, total carbon, total nitrogen, NO_3_^−^–N, and available potassium decreased, but these levels increased after this period [11]. Additionally, related studies indicate a close association between continuous cropping and alterations in the structure of soil microbial communities. Research by Li M et al. demonstrated that with prolonged continuous cropping of melons, the abundance of soil fungi increases, whereas fungal diversity and richness diminish [12]. Cui R et al. noted that compared to non-continuous planting, continuous cropping results in lower bacterial α-diversity in the three subterranean compartments, while the α-diversity of fungi in the roots is higher. There are also significant differences in the composition of fungal communities between the two groups, with the relationship between fungi and environmental factors being closer than that of bacteria [13]. In *T. farfara* production, rhizomes are challenging to completely eradicate following harvest, prompting farmers to often bury them underground for subsequent harvests. Over time, continuous cropping has emerged as a significant constraint and pressing issue in *T. farfara* production. Current research on *T. farfara* primarily concentrates on morphological observations, pharmacological effects, and cultivation practices, while studies addressing its continuous cropping aspects remain limited [6,14]. Consequently, this study focuses on *T. farfara* subjected to continuous cropping for 1, 2, and 3 years, analyzing the yield, quality, and soil physicochemical properties associated with continuous cropping. High-throughput sequencing will be employed to conduct 16S rDNA and ITS sequencing of the microbial communities in the rhizosphere soil of *T. farfara* and to investigate the potential correlations between yield, quality, soil physicochemical properties, and microbial communities in the rhizosphere soil. This study aims to provide a theoretical basis and technical support for understanding the mechanisms of continuous cropping obstacles in *T. farfara* production.

## 2. Materials and Methods

### 2.1. Overview of the Test Area

**Figure 1:**
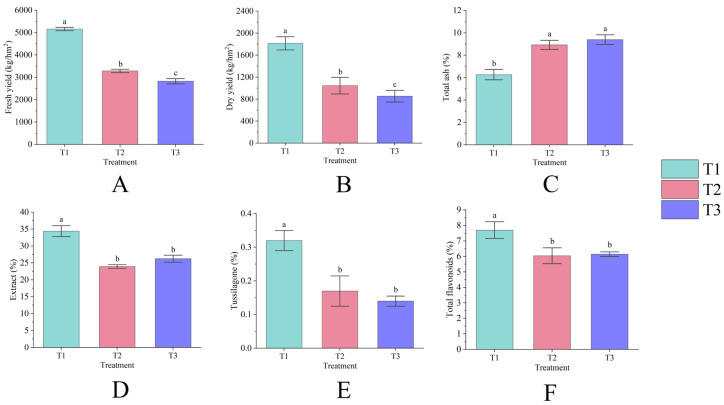
Fresh yield (**A**), dry yield (**B**), total ash (**C**), extract (**D**), tussilagone (**E**), and total flavonoids (**F**) of *T. farfara* flower buds across different consecutive cropping years. Lowercase letters denote significant differences between treatments (*p* < 0.05), and this notation is consistent throughout the text.

The experimental site is situated in Lengkancun, Majiaji Town, Weiyuan County, Gansu Province, China (103°6′47″ E, 35°49′41″ N) (Figure 1A). The average annual temperature at this location is 6.1 °C, accompanied by a frost-free period of 130 days and an annual rainfall of approximately 1000 mm. The elevation of the site is 2350 m, and the soil is classified as mollisol. The initial physicochemical properties of the soil are as follows: pH 7.9, organic matter content of 25.46 g/kg, available nitrogen content of 1.3 g/kg, available phosphorus content of 16.14 mg/kg, and available potassium content of 112.8 mg/kg.

### 2.2. Plant Materials, Reagents and Instruments

The planting material is a high-quality rhizome of *T. farfara* with three nodes, white color, and tender without diseases and pests, which was purchased from the Chinese herbal medicine seedling market in Huichuan Town, Weiyuan County, Dingxi City, Gansu Province, China, and identified as *T. farfara* in the family Asteraceae by Jin Xiaojun, a researcher from the College of Agronomy, Gansu Agricultural University.

The reference standards for high-performance liquid chromatography (HPLC) analysis were procured from Shanghai Yuan Ye Biotechnology Co., Ltd. (Shanghai, China). All standard substances exhibited a purity of 98% or higher. Soil microbial analysis was conducted by Shenzhen Weike Meng Technology Group Co., Ltd. (Shenzhen, China). The reagents used, including isopropanol, acetonitrile, and methanol, were of analytical grade. Ultrapure water was sourced from Hangzhou Wahaha Group Co., Ltd. (Hangzhou, China). The primary instruments utilized in this study comprised the Waters Acquity HPLC system (Waters Corporation, Milford, MA, USA), the Agilent 7890B-5977A gas chromatography–mass spectrometry (GC-MS) system (Agilent Technologies, Santa Clara, CA, USA), and the SCIENTZ-48 high-throughput tissue grinder (Ningbo Xinzhi Biotechnology Co., Ltd., Ningbo, China), among others.

### 2.3. Experimental Design

This experiment employed a single-factor randomized block design, utilizing three plots to cultivate *T. farfara*: one plot that had not been previously planted (T1), one plot that had been planted with *T. farfara* for one year (T2), and one plot that had been planted for two years (T3). All plots were adjacent to each other on a 15° slope, and each plot measured 15 m × 10 m, leading to a total of three treatments, each replicated three times, amounting to nine experimental plots. Prior to planting, a conventional fertilization scheme was implemented, consisting of nitrogen (N: 288 kg/hm^2^), phosphorus (P: 211.5 kg/hm^2^), and potassium (K: 210 kg/hm^2^). This scheme included the application of urea (approximately 46% nitrogen) at a rate of 626 kg/hm^2^, monoammonium phosphate (approximately 48% P and 34% K, expressed as P₂O₅ and K₂O) at 440 kg/hm^2^, and potassium sulfate (approximately 50% K, expressed as K₂O) at 120 kg/hm^2^. The experiment was conducted on 10 April 2022. The direct planting technique involved spacing the rows 25 cm apart, with individual plants spaced 15 cm apart and planting holes dug to a depth of 10 cm. The soil was mulched and harrowed immediately after planting, and 1 m buffer rows were established around each plot. All experimental plots were not subjected to weeding, pesticide application, or other management practices that could potentially affect the soil’s physical and chemical properties, as well as the structure of microbial communities. Other field management practices were maintained consistently across all plots.

### 2.4. Determination of Yield and Quality

#### 2.4.1. Yield Determination

On the morning of 26 November 2022, all *T. farfara* plants from each experimental plot were excavated, and all flower buds within these plots were collected. Following thorough cleaning, the fresh weight of the flower buds was measured to determine the fresh yield of *T. farfara* flower buds for each plot. The collected flower buds were then air-dried at room temperature and weighed to ascertain the dry yield of *T. farfara* flower buds for each plot. The yield per hectare of *T. farfara* flower buds was calculated from the yield obtained from each experimental plot and adjusted according to the area.

#### 2.4.2. Quality Determination

The fully dried *T. farfara* flower buds were ground and sieved. The total ash content and the quantity of ethanol-soluble extract were determined according to the methods outlined in the Pharmacopoeia of the People’s Republic of China (2020 edition). The tussilagone content was quantified using high-performance liquid chromatography (standard curve: y = 27.144x − 73.465, *R*^2^ = 0.997 9). The total flavonoid content of *T. farfara* was measured (standard curve: y = 4.037x − 0.046, *R*^2^ = 0.996 1).

### 2.5. Assessment of the Physicochemical Properties of Rhizosphere Soil and Its Associated Microbial Communities

#### 2.5.1. Collection of Rhizosphere Soil Samples

After harvesting, the rhizosphere soil from plots T1, T2, and T3 was collected. The soil was sampled using a five-point “S” type method, thoroughly mixed in a quadratic manner, and divided into two portions after removing stones and dead leaves. One portion was stored in sterile bags at −80 °C for subsequent microbiological analysis, while the other was allowed to air-dry at room temperature for the determination of soil nutrients and enzyme activity.

#### 2.5.2. Soil Nutrient and Enzyme Activity Determination

Soil pH was measured using the potentiometric method with a water-to-soil ratio of 5:1. Organic matter was quantified by the volumetric potassium dichromate external heating method. Available phosphorus was assessed using the molybdenum–antimony colorimetric method, while available potassium was measured through the ammonium acetate leaching–flame photometric method. Alkaline nitrogen was determined by the alkaline diffusion method. Sucrose enzyme activity was analyzed using the 3,5-dinitrosalicylic acid colorimetric method. Catalase activity was measured via the permanganate titration method. Urease activity was quantified using the phenol-sodium hypochlorite colorimetric method, and alkaline phosphatase activity was assessed with the sodium benzyl phosphate colorimetric method.

#### 2.5.3. Sample DNA Extraction and High-Throughput Sequencing

The composition and diversity of rhizosphere soil bacterial and fungal communities were determined by 16S rDNA and ITS amplicon sequencing techniques [15,16,17]. The methods are as follows.

Total genomic DNA was extracted from rhizosphere soil samples using the CTAB method. The concentration and purity of the DNA were assessed using 1% agarose gels. Based on the concentration, the DNA was diluted to 1 ng/µL with sterile water. Specific primers 341F (5′-CCTAYGGGRBGCASCAG-3′) and 806R (5′-GGACTACNNGGGTATCTAAT-3′) were selected for amplifying the V3 + V4 variable region of the bacterial 16S rRNA gene. Additionally, the ITS1-1F-F (5′-CTTGGTCATTTAGAGGAAGTAA-3′) and ITS1-1F-R (5′-GCTGCGTTCTTCATCGATGC-3′) primers were utilized to target the internal transcribed spacer (ITS) I region of the fungal ribosomal coding gene. PCR amplification was performed using a Bio-Rad T100 thermocycler (Burroughs, St. Louis, Mo, USA). The amplification procedure included a 1 min pre-denaturation step at 98 °C, followed by 30 cycles consisting of 10 s of denaturation at 98 °C, 30 s of annealing at 50 °C, and 30 s of extension at 72 °C, concluding with a 5 min single extension at 72 °C, followed by storage at 4 °C. The PCR mixture consisted of 15 µL of Phusion Master Mix (2×) buffer, 10 µL of gDNA (1 ng/µL), 1 µL of Forward Primer (0.2 µM/µL), 1 µL of Reverse Primer (0.2 µM/µL), and ddH2O to a final volume of 30 μL. PCR reactions were conducted in triplicate, and the resulting products were purified and analyzed using 2% agarose gel electrophoresis (1× TAE). The product purification was carried out using the Qiagen Gum Recovery Kit (Qiagen, Valencia, CA, USA). PE libraries were constructed using Illumina’s TruSeq^®^ DNA PCR-Free Sample Preparation Kit, following the standard protocol provided by Agilent Technologies Co., Ltd. (Santa Clara, CA, USA), and the libraries were sequenced after quantification and an assessment with Qubit. The DNA extraction and PCR amplification were conducted at Shenzhen Weikemen Technology Group Co. (Shenzhen, China), employing the Illumina Novaseq platform (Illumina Experiment Manager 1.19.1, Illumina, headquartered in San Diego, CA, USA) for sequencing analysis.

### 2.6. Data Analysis

The data regarding the flower buds of *T. farfara* and the physicochemical properties of the rhizosphere soil are presented as means ± standard deviations. Data analysis was conducted using IBM SPSS Statistics 26.0, produced by IBM Corp. (Armonk, NY, USA), while graphical representations were created with OriginPro 2021, developed by OriginLab Corporation (Northampton, MA, USA). The raw data were denoised, spliced, and de-chimerized using the QIIME2 system and the qiime tools import program. Subsequent analyses included OTU clustering, alpha diversity, species composition, beta diversity, and species difference analyses. Sequence splicing was conducted with FLASH, sequence filtering was performed using QIIME, and clustering was executed with UPARSE pipeline software. IBM SPSS Statistics 26.0 was utilized for multiple comparisons and significance analysis, while Excel 2019, developed by Microsoft Corporation (Redmond, WA, USA), was employed for statistical organization and graphical representation of the data. The correlation analysis section was executed using OriginPro 2021 (OriginLab, MA, USA) and Chiplot (www.chiplot.online/ accessed on 22 April 2023).

## 3. Results

### 3.1. The Yield and Quality of T. farfara Flower Buds

Continuous cropping significantly impacts the yield of *T. farfara* flower buds (*p* < 0.05), exhibiting a declining trend each year as the duration of continuous cropping increases. Among the treatments, T1 demonstrated the highest yield, with a fresh yield of 5159.53 kg/hm^2^ (Figure 1A) and a dry yield of 1813.91 kg/hm^2^ (Figure 1B). The fresh and dry yields of T2 decreased by 36.33% and 42.35%, respectively, compared to T1, while T3 reflected reductions of 45.26% and 52.88%, respectively, with both significantly differing from T1 (*p* < 0.05). Continuous cropping also substantially influences the quality of *T. farfara* flower buds (*p* < 0.05), as evidenced by an annual increase in total ash content (Figure 1C) and a decrease in tussilagone content each year (Figure 1E). The content of extract (Figure 1D) and total flavonoids (Figure 1F) exhibited a pattern of initial decrease followed by an increase; however, T2 and T3 showed significant differences from T1 (*p* < 0.05), while the differences between T2 and T3 were not significant. The values for each quality indicator for T1 were 6.27%, 34.39%, 0.32%, and 7.70%, while the total ash content for T2 and T3 increased by 42.37% and 49.79%, respectively, compared to T1. In T2 and T3, the contents of extract, tussilagone, and total flavonoids decreased by 30.39%, 48.07%, 21.55% and 24.86%, 57.34%, and 20.28%, respectively, compared to T1.

### 3.2. Physicochemical Properties and Enzymatic Activity of Rhizosphere Soil

**Figure 2:**
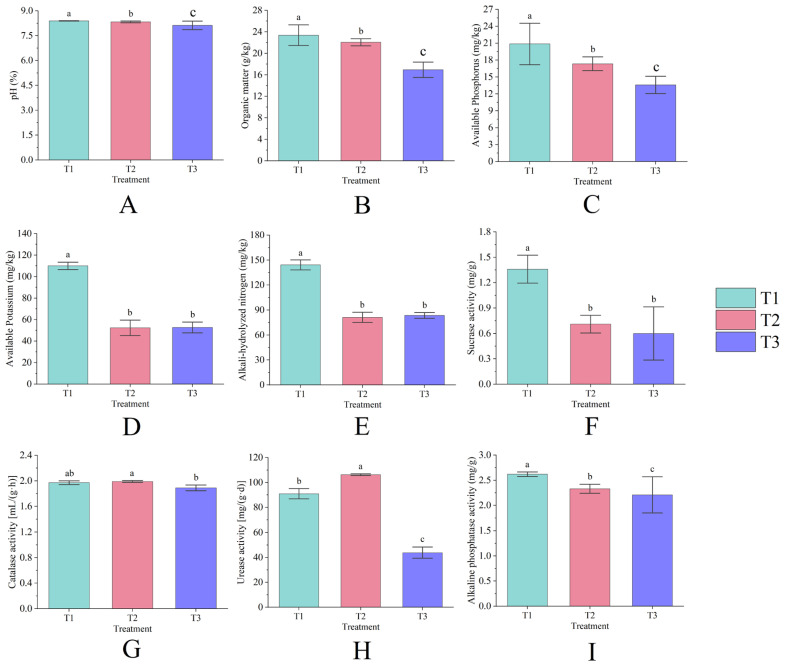
The pH (**A**), organic matter (**B**), available phosphorus (**C**), available potassium (**D**), alkali-hydrolyzed nitrogen (**E**), sucrose activity (**F**), catalase activity (**G**), urease activity (**H**), and alkaline phosphatase activity (**I**) of rhizosphere soil under different years of continuous cropping. Lowercase letters denote significant differences between treatments (*p* < 0.05).

The differences in the content of each nutrient in rhizosphere soil under continuous cropping conditions were statistically significant (*p* < 0.05). The rhizosphere soil Ph (Figure 2A), organic matter (Figure 2B), and available phosphorus (Figure 2C) contents decreased year by year with the increase in continuous cropping years, among which T3 decreased by 3.18%, 27.52%, and 34.85%, respectively, compared with T1; the contents of available potassium (Figure 2D) and alkali-hydrolyzed nitrogen (Figure 2E) showed an overall decrease with the increase in continuous cropping years, T2 decreased by 52.42% and 43.69%, respectively, compared with T1, and T3 was slightly higher than T2, but did not reach a significant level (*p* > 0.05).

The soil sucrase (Figure 2F) and alkaline phosphatase activities (Figure 2I) decreased year by year with the increase in continuous cropping years (*p* < 0.05), and the average annual decline rate reached 54.41% and 8.00%, respectively; catalase activity (Figure 2G) did not show significant changes and remained at 1.89~1.99 mL/(g·h); urease activity (Figure 2H) increased first and then decreased (*p* < 0.05), T2 increased by 16.79% compared with T1, and T3 decreased by 58.75% compared with T2. Among them, both catalase and urease activities increased in the first year of continuous cropping and decreased significantly in the second year of continuous cropping; sucrase and alkaline phosphatase activities were the lowest in the plots in the second year of continuous cropping (*p* < 0.05).

### 3.3. Changes in Rhizosphere Soil Bacterial Community Structure of T. farfara in Different Continuous Cropping Years

#### 3.3.1. Quality Analysis of Rhizosphere Soil Sequencing Results in Different Continuous Cropping Years

**Figure A2:**
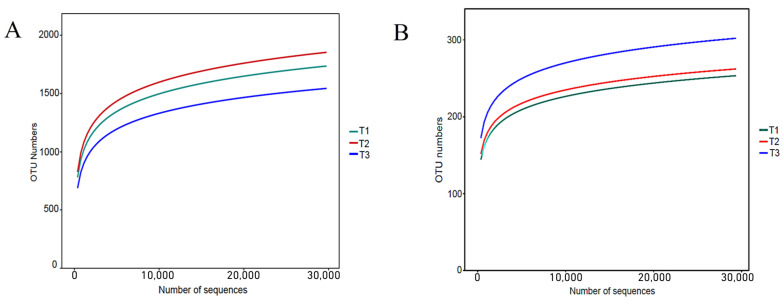
OTU dilution curve of bacteria and fungi in rhizosphere soil of *T. farfara* with different continuous cropping years ((**A**)—Bacteria, (**B**)—Fungi).

As shown in Table A1, 680,542 effective sequences of bacteria were measured in the nine soil samples, of which 425,074 (62.46%) were high-quality sequences with an average length of 430 bp, which were consistent with the bacterial 16S rDNA-V3 + V4 sequences length. The fungal effective sequences were 767,453, of which 582,343 (76.12%) were high-quality sequences with an average length of 261 bp, matching the length of fungal ITS sequences. The dilution curves were able to illustrate the depth of sequencing. Using the observation feature method to plot the dilution curves after categorizing rhizosphere soil bacteria and fungi OTUs with 97% similarity (Figure A2). The results showed that both bacteria and fungi leveled off with increasing sequencing depth, indicating that the sequencing data were close to saturation and the amount of data was reasonable, which could truly reflect the diversity contained in the soil samples.

#### 3.3.2. Distribution of Bacteria and Fungi OUT in Rhizosphere Soil Under Different Years of Continuous Cropping

**Figure 3:**
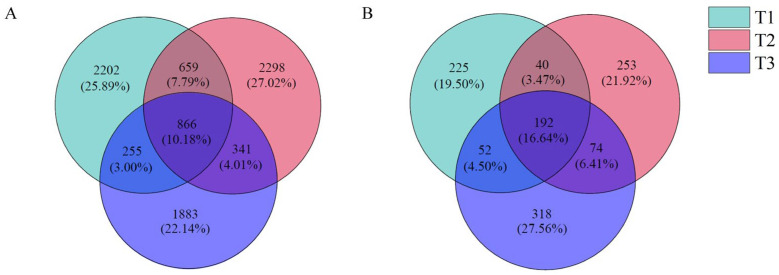
Venn diagram of bacterial (**A**) and fungi (**B**) OTU distribution in rhizosphere soil of *T. farfara* with different continuous cropping years.

The OTU clustering analysis of the sequencing results of the rhizosphere soil bacterial community of *T. farfara* (Figure 3A) showed that the three treatment soil samples had a total of 866 OTUs, accounting for 10.18%; T1, T2, and T3 had 2202, 2298, and 1883 specific OTUs, respectively; T1 and T2 had 659 OTUs (accounting for 7.75%), T1 and T3 had 255 OTUs (accounting for 3.00%), and T2 and T3 had 341 OTUs (accounting for 4.01%).

The OTU clustering analysis of the fungal community sequencing results showed (Figure 3B) that the three treatment soil samples had a total of 192 OUT, accounting for 16.64%; T1, T2, and T3 had specific OTU numbers of 225,253, and 318, respectively; T1 and T2 had a total of 40 OTUs (accounting for 3.47%), T1 and T3 had a total of 52 OTUs (accounting for 4.50%), and 74 OTUs were shared by T2 and T3 (6.41%).

#### 3.3.3. Alpha Diversity Analysis of Rhizosphere Soil Bacteria and Fungi of *T. farfara* in Different Continuous Cropping Years

The Alpha diversity analysis of rhizosphere soil bacterial and fungal communities of *T. farfara* in continuous cropping was conducted by using the Goods_coverage index, Ace index, Chao index, Shannon index, and Simpson index (Table 1); the coverage of bacterial flora detected in all samples reached more than 99%, and Chao index and Ace index increased first and then decreased with the increased of continuous cropping years. The Shannon and Simpson indices showed T2 > T1 > T3, but the differences were not significant (*p* > 0.05). This indicates that the bacterial diversity and abundance of each soil sample increased first and then decreased with the increase in continuous cropping years, and T3 was significantly lower than T1 (*p* < 0.05). On the contrary, the fungal flora coverage detected in all samples reached more than 100% (Table 1), and the community abundance Chao and Ace indices both showed T3 > T2 > T1, which increased with the increase in continuous cropping years, and T3 was significantly different from T1 and T2 (*p* < 0.05); As with the abundance index, both the Shannon and Simpson indices increased with the continuous cropping years, but the changes between T2 and T3 were not significant (*p* > 0.05). It showed that the fungal diversity and abundance of each soil sample increased with the increase in cropping year, and T3 was significantly higher than T1 (*p* < 0.05).

#### 3.3.4. Changes in Rhizosphere Soil Bacteria Community Structure of *T. farfara* in Different Continuous Cropping Years

**Figure 4:**
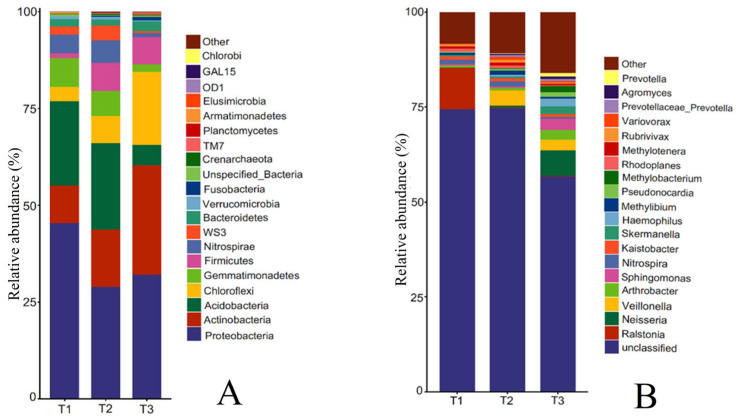
Relative abundance of bacterial species at phylum level and genus level in rhizosphere soil of *T. farfara* with different continuous cropping years ((**A**): phylum level; (**B**): genus level).

The rhizosphere soil bacterial community of *T. farfara* at the phylum level mainly consisted of Proteobacteria, Acidobacteria, Actinobacteria, Gemmatimonadetes, Nitrospirae, Chloroflexi, WS3, Bacteroidetes, and Firmicutes (Figure 4A). The relative abundance of Proteobacteria ranked first, decreasing first and then increasing with the increase in continuous cropping years, which was 45.39%, 28.93%, and 31.99%, respectively. The relative abundance of Acidobacteria, Nitrospirae, and WS3 showed an increase first and then a decrease, but the changes between T1 and T2 were not significant, and T3 decreased by 75.95%, 78.53%, and 74.41%, respectively, compared with T1, reaching significant differences (*p* < 0.05). The relative abundance of Gemmatimonadetes was 7.52%, 6.46%, and 1.95%, respectively, decreasing year by year. In contrast to Gemmatimonadetes, Actinobacteria, Chloroflexi, Bacteroidetes, and Firmicutes showed a yearly increase with the increase in cropping years, and the relative abundance of each phylum in T3 increased by 1.94, 4.22, 0.34, and 4.47 times compared with T1, respectively.

Further analysis of the rhizosphere soil bacterial community at the genus level of *T. farfara* (Figure 4B) showed that the top 10 genera mainly included Ralstonia, Neisseria, Veillonella, Arthrobacter, Sphingomonas, Nitrospira, Kaistobacter, Skermanella, Haemophilus, and Methylibium. With the increase in continuous cropping years, the relative abundance of Ralstonia and Nitrospira decreased among which Ralstonia changed most significantly, T3 decreased by 76.86 times compared with T1. The relative abundance of Arthrobacter, Sphingomonas, and Skermanella increased with the increase in cropping years, and T3 increased by 3.69, 7.74, and 7.08 times, respectively, compared with T1. The relative abundance of Haemophilus, Neisseria, and Veillonella in T1 was less than 0.1%, increased with the increase in cropping years, and T3 increased 167.25 and 68.75 times, respectively, compared with T1. The relative abundance of Methylibium increased first and then decreased with the increase in continuous cropping years, with the highest in T2.

#### 3.3.5. Changes in Rhizosphere Soil Fungal Community Structure of *T. farfara* in Different Continuous Cropping Years

**Figure 5:**
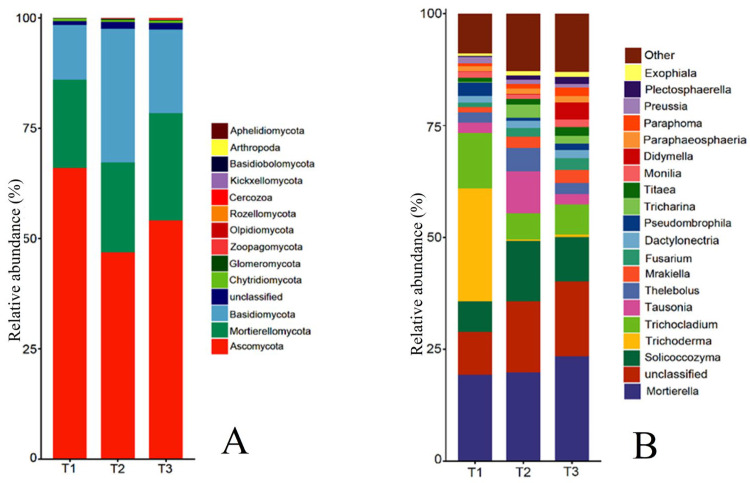
Relative abundance of fungi species at phylum level and genus level in rhizosphere soil of *T. farfara* in different continuous cropping years ((**A**): phylum level; (**B**): genus level).

The rhizosphere soil fungal community at the phylum level mainly consisted of Ascomycota, Mortierellomycota, Basidiomycota, Fungi_unclassified, Chytridiomycota, Zoopagomycota, Glomeromycota, and Olpidiomycota (Figure 5A). The relative abundance of Zygomycota was ranked first and decreased year by year, which was 65.96%, 54.10%, and 46.80%, respectively. The relative abundance of Chytridiomycota and Zoopagomycota was 0.58%, 0.42%, and 0.49%, and 0.08%, 0.06%, and 0.21%, respectively, which also showed a decrease first and then increase with the increase in cropping years, and the relative abundance of Chytridiomycota was not significant in T1, T2, and T3, while Zoopagomycota increased 162.27% in T3 compared with T1. The relative abundance of Mortierellomycota, Fungi_unclassified, and Olpidiomycota increased gradually with the increase in cropping years, reaching 24.29%, 1.61%, and 0.20% in T3, respectively, and the relative abundance of Olpidiomycota changed the most, increasing 5.67 times in T3 compared with T1. The relative abundance of Basidiomycota and Glomeromycota was the opposite of Ascomycota, showing an increase first and then a decrease with the increase in cropping years, with the relative abundance of 12.41%, 30.19%, 18.89%, and 0.04%, 0.28%, 0.14%, respectively, all reaching the highest in T2.

The top 20 genera of rhizosphere soil fungal communities of *T. farfara* include Mortierella, Trichoderma, Solicoccozyma, etc. (Figure 5B). Among them, the relative abundance of Trichoderma and Didymella changed the most with the increase in cropping years, and the relative abundance of Trichoderma decreased from 25.24% in T1 to 0.61% in T3, which decreased by 40.38 times; the relative abundance of Didymella increased from 0.07% in T1 to 3.80% in T3, which increased by 53.29 times, indicating that Trichoderma and Didymella have a higher sensitivity to continuous cropping. The relative abundance of Mortierella, Unspecified_Nectriaceae, Mrakiella, Fusarium, Unspecified_Didymellaceae, Titaea, Unspecified_Fungi, and Paraphoma increased with the increase in cropping years, with T3 increased by 21.30%, 92.20%, 157.39%, 168.75%, 916.67%, 124.14%, 75.58%, and 196.97%, respectively, compared with T1, and all differed significantly from T1 (*p* < 0.05), with the relative abundance of Unspecified_Didymellaceae changing nearly 10 times. In contrast to the Mortierella, the relative abundance of the Trichocladium and Pseudombrophila in T1 was significantly higher than in T2 and T3, but the variation between T2 and T3 did not reach significance. The relative abundance of Solicoccozyma, Tausonia, Thelebolus, Unspecified_Sebacinales, and Tricharina all showed increases first and then decreases with the increase in continuous cropping years, but each genus was still significantly higher than T1 after the decrease.

#### 3.3.6. Beta Diversity Analysis of Rhizosphere Soil Bacteria and Fungi in Different Continuous Cropping Years

**Figure 6:**
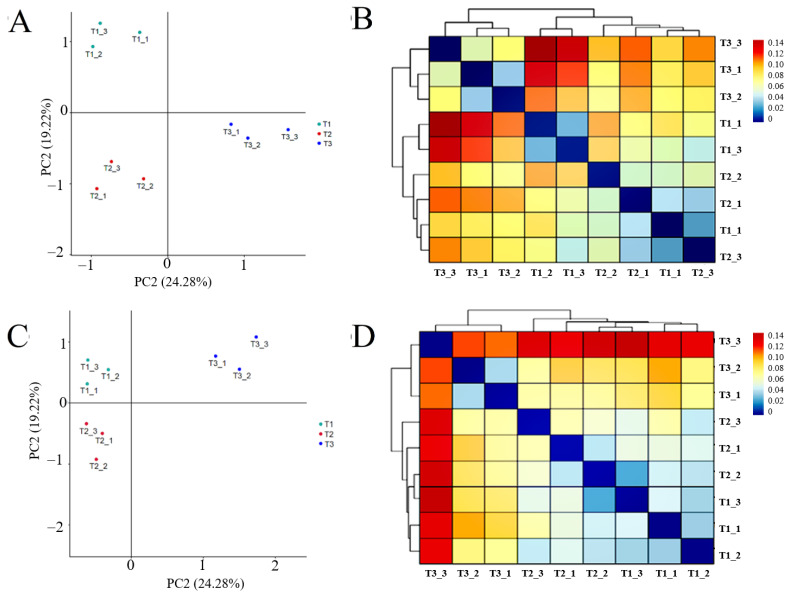
β-diversity-PCA analysis and Weighted Unifrac heat map of bacterial and fungal community in rhizosphere soil of *T. farfara* with different continuous cropping years ((**A**,**B**) are bacterial, (**C**,**D**) are fungi).

PCA analysis of rhizosphere soil bacteria and fungi sequencing results showed (Figure 6A,B) that the structure of rhizosphere soil bacterial and fungal communities in different continuous cropping years of *T. farfara* was zoned without overlapping parts, and the heat map of cluster analysis based on β-diversity-Weighted Unifrac distance showed (Figure 6C,D) that the bacterial and fungal communities in T1 and T2 were more similar and clustered into one group, while T3 clustered into one group alone, indicating that the microbial community structure among rhizosphere soil in different continuous cropping years differed significantly, and the differences between T1 and T2 were small and became larger with the increase in continuous cropping years.

### 3.4. Correlation Analysis Among the Yield and Quality of Continuous Cropping of T. farfara: Flower Buds, Soil Physicochemical Properties, and Rhizosphere Soil Microorganisms

**Figure 7:**
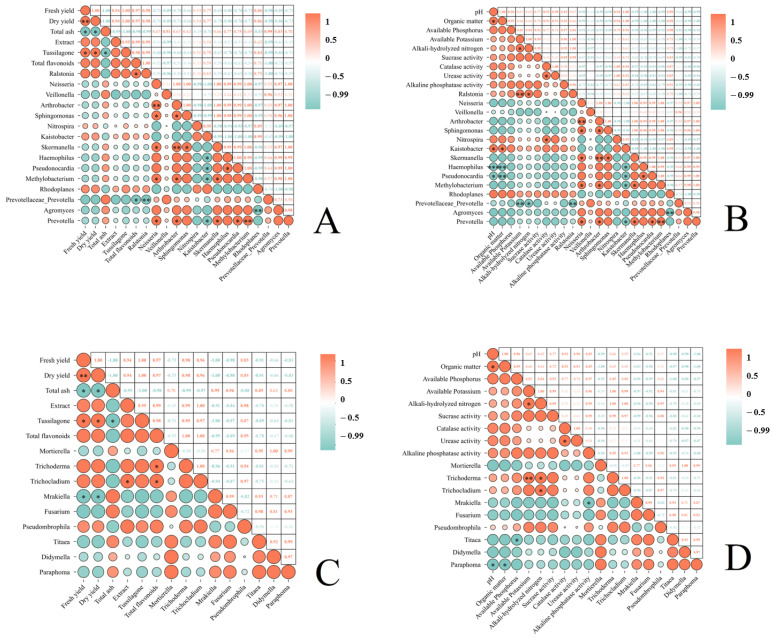
Analyzing the correlation between the relative abundance of different microbial genera in the rhizosphere soil subjected to various treatments and the yield and quality of *T. farfara* flower buds, as well as the soil’s physicochemical properties. (**A**): Correlation analysis between the yield and quality of *T. farfara* flower buds and the abundance of bacteria in the rhizosphere soil; (**B**): correlation analysis between the physicochemical properties of soil under continuous cropping of *T. farfara* and the abundance of bacteria in the rhizosphere soil; (**C**): correlation analysis between the yield and quality of *T. farfara* flower buds and the abundance of fungi in the rhizosphere soil; (**D**): correlation analysis between the physicochemical properties of soil under continuous cropping of *T. farfara* and the abundance of fungi in the rhizosphere soil. Asterisks denote significance levels: *** *p* < 0.001; ** *p* < 0.01; * *p* < 0.05.

Through a series of correlation analyses (Figure 7), we obtained the following results: Total flavonoids in *T. farfara* flower buds exhibited a significant negative correlation with Prevotellaceae_Prevotella (*p* < 0.05) (Figure 7A). In soils subjected to continuous cropping of *T. farfara*, pH and organic matter showed a significant positive correlation with Kaistobacter (*p* < 0.05), a significant negative correlation with Pseudonocardia (*p* < 0.05), and a highly significant negative correlation with Haemophilus (*p* < 0.01). Available potassium was highly positively correlated with Ralstonia (*p* < 0.01) and highly negatively correlated with Prevotellaceae_Prevotella (*p* < 0.01). Alkali-hydrolyzed nitrogen demonstrated a significant positive correlation with Ralstonia and a significant negative correlation with Prevotellaceae_Prevotella (*p* < 0.05). Catalase activity was significantly positively correlated with Nitrospira (*p* < 0.05) (Figure 7B). In *T. farfara* flower buds, the extract showed a significant positive correlation with Trichocladium, and total flavonoids exhibited a significant positive correlation with both Trichoderma and Trichocladium (*p* < 0.05). Fresh yield and dry yield were significantly negatively correlated with Mrakiella (*p* < 0.05) (Figure 7C). In soils under the continuous cropping of *T. farfara*, pH and organic matter displayed a significant negative correlation with Paraphoma (*p* < 0.05), available phosphorus was significantly negatively correlated with Titaea, available potassium was highly positively correlated with Trichoderma (*p* < 0.01), alkali-hydrolyzed nitrogen had a significant positive correlation with Trichoderma and Trichocladium (*p* < 0.05), and alkaline phosphatase activity was significantly negatively correlated with Mrakiella (*p* < 0.05).

## 4. Discussion

### 4.1. Continuous Cropping of T. farfara Has Adverse Effects on the Yield and Quality of Its Flower Buds

Continuous cropping negatively impacts both the yield and quality of crops. This practice leads to the accumulation of harmful substances secreted by plant roots in the soil, resulting in autotoxicity [18,19,20]. Furthermore, prolonged cultivation depletes soil nutrients, which consequently affects the growth and development of crops [21]. This study demonstrated that an increase in the duration of continuous cropping of *T. farfara* leads to a significant decline in fresh yield, dry yield, extract, tussilagone, and total flavonoid content (*p* < 0.05). Simultaneously, a significant increase in total ash content was observed (*p* < 0.05). These results indicate that continuous cropping adversely affects both the yield and quality of *T. farfara*, with the negative effects becoming more pronounced over longer cropping periods. This is consistent with the findings of Ku Y, Fujisao K, and others [9,10].

### 4.2. Continuous Cropping of T. farfara Has Adverse Effects on the Soil Environment

Continuous cropping degrades soil quality and modifies the soil environment, thereby adversely impacting crop growth and resulting in diminished plant vigor, reduced yield, and lower quality [22,23]. Moreover, crops become increasingly vulnerable to pests and diseases due to decreased resistance [24]. This study demonstrated that, with the prolonged duration of the continuous cropping of *T. farfara*, there was a significant decline in pH, organic matter, available phosphorus, available potassium, alkaline nitrogen content, sucrose enzyme activity, catalase activity, urease activity, and alkaline phosphatase activity (*p* < 0.05). These results indicate that the continuous cropping of *T. farfara* not only diminishes soil nutrients and reduces enzyme activity but also leads to soil acidification, with these effects becoming more pronounced with extended cropping durations. This aligns with the findings of Zhang Y, Wang Y, and others [25,26].

### 4.3. Increasing Continuous Cropping Years Affect Rhizosphere Soil Bacterial Diversity and Community Structure of T. farfara

Soil microorganism is an important part of maintaining soil structure and guaranteeing the stability of micro-ecosystems. They function through different microflora to maintain ecosystem stability, which is one of the factors affecting normal plant growth and development, and there are significant differences in microbial community structure between different soils or different plots of the same soil type [27,28,29]. The diversity of the rhizosphere soil bacterial community has been used as an important indicator to assess soil quality and fertility and plays an important role in regulating soil fertility, nutrient distribution, and disease suppression [30,31].

In this study, the analyzed rhizosphere soil samples from different continuous cropping years of *T. farfara* revealed that the α-diversity of the bacterial community increased first and then decreased with the increase in continuous cropping years, and the bacterial abundance and diversity index in soil samples from continuous cropping for two years were the smallest and significantly lower than the rotational cropping, which was consistent with the results of Li J. [32]. Comparison of bacterial composition and abundance in rhizosphere soil of different continuous cropping years showed that the main dominant bacterial phyla in the samples were all Proteobacteria, Actinobacteria, and Acidobacteria, but the relative abundance of each phylum varied greatly among treatments. Proteobacteria and Actinobacteria are beneficial in soil, which can help plant growth and participate in the carbon and nitrogen cycle, and also can effectively degrade pesticide residues in soil and restore the soil environment. In this study, the relative abundance of Proteobacteria was significantly lower than the rotational cropping, which was consistent with the changes in Chrysanthemum morifoliumunder continuous cropping, indicating that the Proteobacteria bacteria are highly adaptable to the changes in soil environment under continuous cropping conditions [32]. Unlike Proteobacteria, the relative abundance of Actinobacteria increased with the increase in continuous cropping years, presumably because the soil environment was still suitable for their growth and reproduction during the short continuous cropping years, and might decrease with the extension of cropping years. It has been shown that Acidobacteria can provide nutrients to the soil by degrading complex lignin and cellulose [33]. In this study, the relative abundance of Acidobacteria decreased gradually with the increase in cropping years, indicating that the change in soil environment under continuous cropping would inhibit the growth and reproduction of Acidobacteria, and the energy provided by soil microorganisms would decrease. Among the top 10 genera, the relative abundance of Ralstonia and Nitrospira decreased significantly with the increase in continuous cropping years. As environment-friendly microorganisms for the degradation of benzene substitute pollutants and PHB synthesis, Ralstonia has great potential and advantages in environmental pollution management. Nitrospira is often found in neutral or weakly alkaline environments as nitrifying bacteria in soil, which can oxidize nitrite to nitrate in soil and promote nitrogen cycle; Neisseria bacteria can ferment a variety of sugars and produce acid but not gas; Veillonella bacteria can participate in fermentation metabolism, fermenting pyruvic acid, oxalic acid, malic acid, and producing acetic acid, propionic acid, CO_2_, and H_2_ [34]. Therefore, the relative abundance of Nitrospira decrease with the increase in continuous cropping years may be related to the decrease in soil pH due to acid production by bacteria of the genera Neisseria and Veillonella. Nitrifying bacteria can directly oxidize and utilize ammonia, which will be converted to ammonium under acidic conditions, thus making it impossible for nitrifying bacteria to survive. Sphingomonas can degrade a variety of macromolecular organic compounds and is resistant to a variety of pesticides and herbicides [35]. The relative abundance of Sphingomonas in rhizosphere soil significantly increases in continuous cropping for two years may be related to the use of the resistance of pesticides in the previous planting process that has made them resistant. The PCA analysis showed that the soil bacterial communities in different quadrants and far apart in different continuous cropping years, indicating that there were significant differences in bacterial community structure with increasing cropping years. Meanwhile, the number of rhizosphere soil bacteria OUT decreased significantly by 16.00% compared with the rotational cropping after continuous cropping for two years, indicating that the number of bacteria decreased significantly after continuous cropping for two years, which was consistent with the clustering analysis in which the samples were grouped into one category, This indicates that the rhizosphere soil bacterial community did not change significantly in the early stage, but the number decreased and the structure of the bacterial community changed with the extension of the continuous cropping years.

### 4.4. Increasing Continuous Cropping Years Affect the Rhizosphere Soil Fungi Diversity and Community Structure of T. farfara

Soil microbial balance serves as a key indicator of soil health. Soil fungi significantly influence soil health and are vital components of the soil microbiome, contributing to considerable diversity in both abundance and species. In conjunction with other microorganisms, they play a crucial role in maintaining the balance and stability of the entire ecosystem while participating in material cycling and energy flow [36]. In this study, it was found that continuous cropping of *T. farfara* disrupted the soil microecological balance and changed the fungal diversity, and the number of fungal species in rhizosphere soil increased with the increase in continuous cropping years, especially the number of fungal species reached 180 in two years of continuous cropping, which was 22.45% higher than the rotational cropping. The α-diversity analysis of rhizosphere soil fungal communities in different cropping years showed that both abundance and diversity indices increased with the increase in continuous cropping years.

The differences in cropping growth and cropping years will lead to changes in the soil microcosm environment, fungal community structure, composition, and abundance [11,12,13]. In this study, we found that the main dominant fungal phyla in the rhizosphere soil of *T. farfara* were the same and remained stable, with the highest relative abundance of Ascomycota (46.80–65.69%), followed by Mortierellomycota (20.00–24.29%) and Basidiomycota (12.41–18.89%). Most of the Ascomycota are nutrient-dependent fungi, while most of the Basidiomycota are nutrient-poor fungi that grow and reproduce more slowly, with both being associated with organic matter degradation [37]. When the soil nutrients and enzyme activity is gradually decreased after continuous cropping, the relative abundance of Ascomycota is decreased, which is sensitive to the soil environment. Because of the slow growth and reproduction of Basidiomycota, soil nutrients are still sufficient after one year of continuous cropping, in the same time the cropping growth process will secrete a lot of metabolic substances to provide nutrients for Basidiomycota, but with the increase of continuous cropping years, the supply of soil nutrients is insufficient and the organic matter content decreases; therefore, the relative abundance of Basidiomycota showed an increase and then decrease after continuous cropping. Among the top 10 fungal genera, the relative abundance of Mortierella and Fusarium increased with the increase of continuous cropping years. It has been shown that Mortierella is endophytic fungi of many plant roots, and most of them are harmful which can cause plant and animal diseases and cause some losses in production [38]. Fusarium is relatively large among fungi, and can cause plant wilt, root rot through infestation, evolving into wilt and root rot, which seriously affects production and profitability [39]. Among them, Fusarium acuminatum, which is a fungus belonging to the genus Fusarium, is a major disease in the production of *T. farfara*, causing the plant to wilt and die [40]. The relative abundance of Fusarium in the rhizosphere soil of continuous cropping for two years was 1.69 times higher than the rotational cropping. Ku Y et al. showed that the imbalance of microbial community structure due to the massive accumulation of pathogenic bacteria is the core mechanism causing continuous cropping obstacles [9]. Trichoderma can produce bioactive substances that are antagonistic to a variety of plant pathogens, regulating plant growth, development, and metabolism through nutritional competition and microparasitism, which plays an important role in ensuring the structural balance of soil microbial communities [41,42]. In this study, the relative abundance of Trichoderma decreased significantly with the increase of continuous cropping years, and the relative abundance of this genus in the rhizosphere soil continuous cropping for two years decreased by 40.38 times compared with the rotational cropping, indicating that the structure of the *T. farfara* fungal community has been imbalanced under continuous cropping conditions, and Trichoderma could not play a corresponding role.

## 5. Conclusions

In comparison to T1, the T2 and T3 treatments negatively impact the yield and quality of *T. farfara* flower buds, as well as the physicochemical properties of the rhizosphere soil and the associated microbial community. Continuous cropping leads to a decline in both the yield and quality of *T. farfara* flower buds, while also degrading the soil environment. This results in a reduction of soil nutrients and gradual acidification. Moreover, continuous cropping disrupts the rhizosphere microbial community. The alterations in the microbial community are closely related to the physicochemical properties of the soil and the yield and quality of *T. farfara* flower buds. Future research should prioritize understanding the specific mechanisms underlying these impacts and exploring potential methods for artificially intervening to regulate the soil microbial community, thereby improving the quality of soil subjected to continuous cropping and enhancing crop yield and quality.

## Figures and Tables

**Table 1:** Alpha diversity of bacteria and fungi in rhizosphere soil of *T. farfara* with different continuous cropping years.

Treatment	Bacteria			Fungi		
	T1	T2	T3	T1	T2	T3
Goods_coverage	0.99 a	0.99 a	0.99 a	1.00 a	1.00 a	1.00 a
Chao	1820.06 b	1966.82 a	1628.20 c	248.67 b	255.67 b	296.00 a
Ace	1821.88 b	1970.68 a	1632.24 c	272.00 b	280.00 b	322.00 a
Shannon	9.24 ab	9.88 a	8.92 c	4.41 b	5.24 a	5.69 a
Simpson	0.98 a	0.99 a	0.98 a	0.78 b	0.93 a	0.95 a

Lowercase letters denote significant differences between treatments (*p* < 0.05).

**Figure A1:**
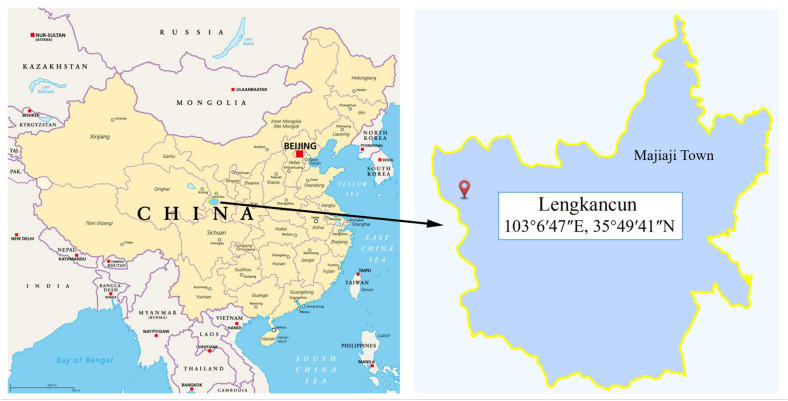
Schematic diagram of the test site.

## Data Availability

Any data mentioned in this article can be obtained by contacting the corresponding author.

## Appendix A

**Table A1:** High-quality sequence statistics of rhizosphere soil in different continuous crop ping years.

Treatment	Bacteria			Fungi		
	Valid Sequence	High-Quality Sequence	Proportion/%	Valid Sequence	High-Quality Sequence	Proportion/%
T1	72,289	44,243	61.27	86,236	65,999	76.44
T2	80,784	51,961	63.81	83,916	65,380	78.06
T3	73,774	45,487	60.97	85,665	62,736	73.88
Total	680,542	425,074	62.46	767,453	582,343	76.12
